# Association between 16S rRNA variation patterns and *mgpB* phylogeny in *Mycoplasma genitalium*

**DOI:** 10.1128/spectrum.00918-26

**Published:** 2026-05-19

**Authors:** Kazuo Imai, Ryuha Omachi, Akihiro Sato, Masashi Tanaka, Nobuaki Mori, Takuya Maeda

**Affiliations:** 1 Department of Clinical Laboratory Medicine, Saitama Medical University 681431, Iruma Gun, Saitama, Japan; 2 Department of Infectious Disease and Infection Control, Saitama Medical University 13031, Iruma Gun, Saitama, Japan; 3 KARADA Internal Medicine Clinic, Shinagawa City, Tokyo, Japan; 4 Department of Medicine, Division of Clinical Infectious Diseases, Showa Medical University School of Medicine 38557 , Shinagawa City, Tokyo, Japan; Houston Methodist Hospital, Houston, Texas, USA

**Keywords:** drug resistance, *Mycoplasma genitalium*, 16S rRNA

## LETTER

*Mycoplasma genitalium* is an important sexually transmitted pathogen. Sequence variations in the 16S rRNA gene have been reported in several studies of the *Mycoplasma* genus, particularly in regions corresponding to helix 31 (H31) and helix 34 (H34) based on *Escherichia coli* numbering, which include the primary tetracycline binding site (1–4). These regions have been the focus of exploratory analyses, although their link to tetracycline resistance has not been established in *M. genitalium* (4). Studies from Germany (5), France (6, 7), and Australia (4) have identified sequence variations at positions GG966–967TT in H31, with detection rates of 4.5%–13.5%, whereas a study from China reported C1192T near H34 in 22.5% of isolates (8). Variations outside these regions have also been described, including A746G and C1460T, with variable prevalence across geographic regions. These findings suggest regional differences in 16S rRNA variation, but it is unclear whether they reflect specific phylogenetic lineages. Furthermore, the distribution of 16S rRNA variations in Japanese *M. genitalium* samples have not been described. The present study aimed to characterize these variations and their relationship with *mgpB*-defined phylogenetic lineages.

The *mgpB*, *MG309*, and 16S rRNA genes were amplified by PCR and sequenced (4, 9–11). Primers were designed outside the 16S rRNA gene to obtain full-length sequences, and genotyping was performed using the PubMLST database. Longitudinal samples with identical genotypes from the same patients were excluded. This study was approved by the Institutional Review Board of Saitama Medical University (2023-026), with patient consent obtained via an opt-out procedure.

**Fig 1:**
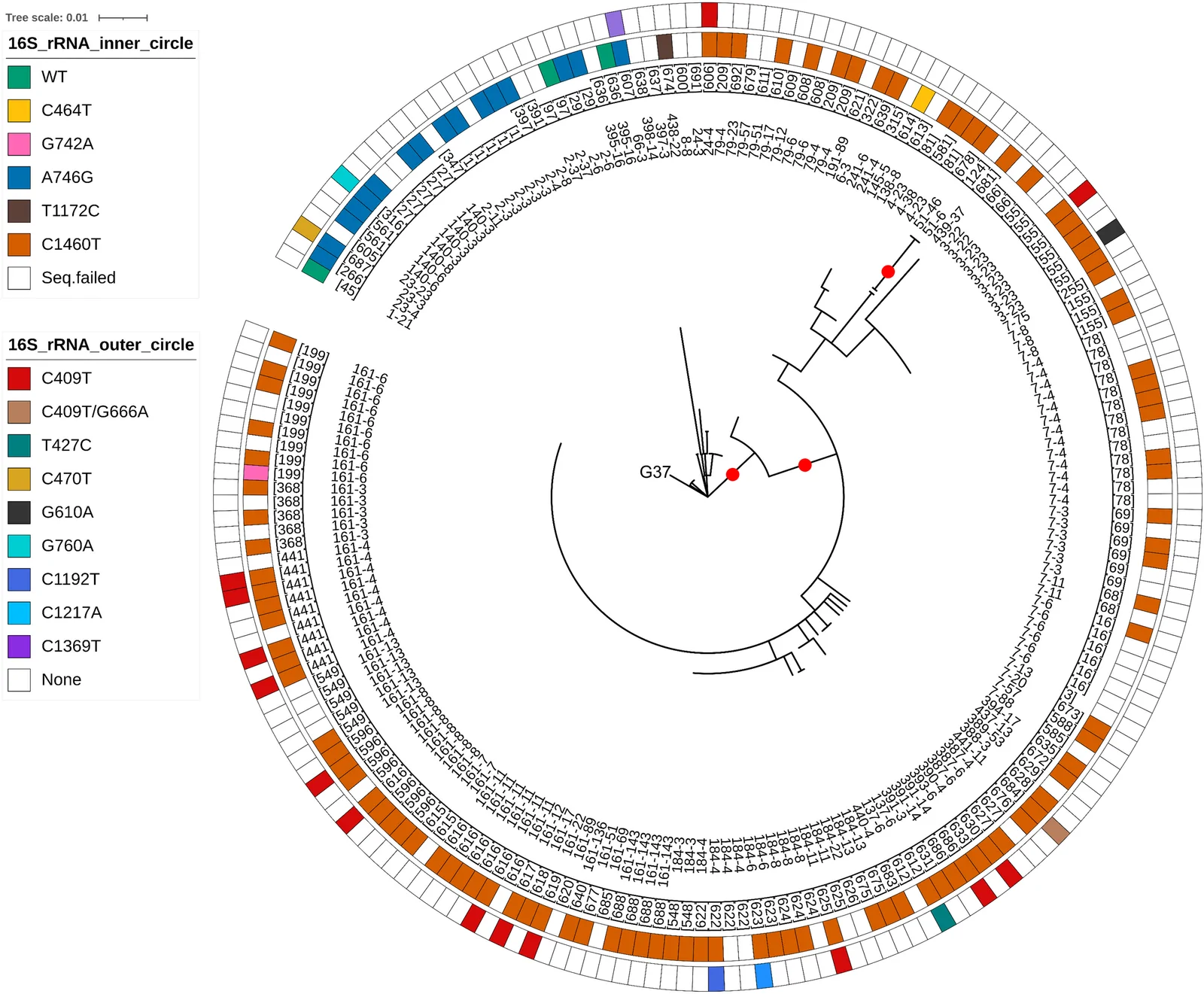
Phylogenetic tree of *mgpB* with 16S rRNA variation patterns. A phylogenetic tree was constructed using the maximum likelihood method in IQ-TREE (*n* = 189) and was rooted with the reference strain G37 (NC_000908). From the inner to outer circles, the rings represent *mgpB–MG309* alleles based on PubMLST, sequence types, and 16S rRNA variation patterns. Branches with ultrafast bootstrap values ≥ 95% are indicated by red circles. 16S rRNA variant positions are based on *E. coli* numbering. The inner circle indicates the first 16S rRNA variant in each sample, whereas the outer circle indicates additional co-occurring variants. The branch corresponding to *mgpB* allele 51 was truncated to improve visibility.

Among 188 samples (male urine, *n* = 146; female vaginal swabs, *n* = 42) with *mgpB* and *MG309* genotyping, 16S rRNA sequencing was successful in 121 samples (median age, 30 years; 97 [80.2%] male; and 116 [95.9%] symptomatic). Most of these patients (108, 89.3%) received fluoroquinolone monotherapy or tetracycline followed by a fluoroquinolone, whereas 4 (2.5%) received tetracycline monotherapy. Data on sexual networks and prior antibiotic exposure were not collected. The most frequent variants outside H31 and H34 were C1460T (99/121, 81.8%), C409T (16/121, 13.2%), and A746G (16/121, 13.2%), with all C409T strains also carrying C1460T. Only one sample (0.8%) harbored the C1192T variant in H34 (Table 1). Phylogenetic analysis revealed a clear association between *mgpB*-defined clusters and 16S rRNA variation patterns outside H31 and H34 (Fig. 1). The clusters were broadly divided into those with wild-type or A746G sequences and those predominantly carrying C1460T or C409T/C1460T variants.

**TABLE 1:** Distribution of 16S rRNA variants in *M, genitalium* (*n* = 121)^*a*^

*M. genitalium* numbering	*E. coli* numbering	*n* (%)
Wild-type (G37)		3 (2.5)
C404T^a^	409	16 (13.2)
T423C^a^	427	1 (0.8)
C462T	464	1 (0.8)
C468T	470	1 (0.8)
G607A^a^	610	1 (0.8)
G662A^b^	666	1 (0.8)
G738A	742	1 (0.8)
A742G	746	16 (13.2)
G756A^c^	760	1 (0.8)
T1147C	1172	1 (0.8)
C1166T^a^	1192	1 (0.8)
C1191A^a^	1217	1 (0.8)
C1343T^c^	1369	1 (0.8)
C1435T	1460	99 (81.8)

^*a*^ The variant was detected concomitantly with C1435T^(a)^, C404T/C1435T^(b)^, or A742G^(c)^; positions are based on *M. genitalium* numbering. The 16S rRNA sequences of *E. coli* K-12 (NC_000913) and *M. genitalium* G37 (NC_000908) were used for alignment, which was performed using MEGA (ver. 7.0). The 16S rRNA sequences generated in this study have been deposited in GenBank (accession numbers LC921957–LC922077).

These findings indicate that A746G, C1460T, and the newly identified C409T represent lineage-associated variants. Regional differences in 16S rRNA variants likely reflect local genotype distribution rather than independent emergence. This study is the first to characterize 16S rRNA variation in Japanese clinical samples. Ongoing surveillance is needed to monitor temporal shifts in the prevalence of these variants and explore their associations with phylogenetic lineages. However, susceptibility testing, tetracycline treatment outcomes, and the impact of prior antibiotic exposure were not available, precluding assessment of the functional or clinical significance of these variants. Future studies should assess links between these variants and tetracycline treatment failure.
